# Mechanisms of Aortic Flow Deceleration and the Effect of Wave Reflection on Left Ventricular Function

**DOI:** 10.3389/fphys.2020.578701

**Published:** 2020-11-05

**Authors:** Chloe M. Park, Alun D. Hughes, Michael Y. Henein, Ashraf W. Khir

**Affiliations:** ^1^Brunel Institute for Bioengineering, Brunel University London, London, United Kingdom; ^2^Institute of Cardiovascular Science, University College London, London, United Kingdom; ^3^Umea Heart Centre, Umea University Hospital, Umea, Sweden

**Keywords:** wave reflection, LV velocity of axis shortening, aortic flow deceleration, wave intensity analysis and forward decompression wave, wave intensity analysis, aortic flow

## Abstract

Increased wave reflection is an independent predictor of cardiovascular events, possibly due to effects on left ventricular (LV) function. We investigated the relationship between reflected waves in early systole, the forward decompression wave in mid-late systole and LV mechanical behavior. Invasively acquired ascending aortic velocity, pressure, and LV long and minor axes’ dimensions were measured simultaneously in 11 anesthetized dogs during both control conditions and aortic occlusion to cause additional early wave reflection. Wave intensity analysis (WIA) was used to identify the arrival of the reflected wave and the onset of a forward decompression wave in mid-late systole. The arrival time of the reflected wave coincided with the time when minor axis shortening began to decline from its peak, even during aortic occlusion when this time is 12 ms earlier. The initial decline in long axis shortening corresponded to the time of the peak of the reflected wave. The forward decompression wave was consistently observed to have a slow and then rapid phase. The slow phase onset coincided with time of maximum shortening velocity of the long axis. The onset of the later larger rapid phase consistently coincided with an increased rate of deceleration of both axes during late systole. Forward decompression waves are generated by the LV when the long axis shortening velocity falls. Reflected wave arrival has a detrimental effect on LV function, particularly the minor axis. These observations lend support to suggestions that therapies directed toward reducing wave reflection may be of value in hypertension and cardiovascular disease.

## Introduction

Increased wave reflection is an independent predictor of cardiovascular events in hypertension (London et al., 2001; Manisty et al., 2010) and has been proposed as a therapeutic target in cardiovascular disease (O’Rourke and Safar, 2005). Reflected waves arrive at the left ventricle (LV) during systole (Baksi et al., 2009) and impose an additional load on the LV, which is exaggerated in hypertension (Merillon et al., 1983; Westerhof and O’Rourke, 1995) and heart failure (Curtis et al., 2007).

LV motion undergoes a complex pattern of change during the cardiac cycle (Codreanu et al., 2011) however, no studies have simultaneously measured the arrival of reflected waves during systole with LV long and minor axes function. Systolic LV function involves shortening of myocardial fibers oriented in circumferential, longitudinal, and oblique directions (Greenbaum et al., 1981; Henein and Gibson, 1999). This arrangement optimizes work (Torrent-Guasp et al., 2005) and has a major influence on intramural stress distribution (Arts et al., 1991). Velocity and strain patterns of various myocardial components have provided valuable information on cardiac function (Nagueh et al., 1997; Wang et al., 2005); however, the relationship between arterial wave reflection, LV mechanical function, and aortic flow remains modestly understood.

Wave intensity analysis (WIA) has proved a useful method for studying pressure-flow dynamics in conjunction with LV wall motion patterns in health and disease (Parker, 2009). Rapid shortening of the LV muscle fibers generates a large forward compression wave (FCW) in early systole, and this has been proposed as an indicator of LV systolic function (Parker et al., 1988). A backward compression wave (BCW) occurring after the initial FCW wave is attributed to reflection of the forward wave from distal sites of impedance mismatch (Zambanini et al., 2002). The origin of the late systolic (proto-diastolic) forward decompression wave (FDW) that decelerates aortic blood flow (Parker et al., 1988) is less well understood.

We hypothesized that wave reflections would influence LV minor (M) and long (L) axis function. Further, to establish mechanistic links between waves and LV function, we used aortic occlusion as means of inducing earlier and larger wave reflections and examined their effect on LV function.

## Materials and Methods

The experimental protocol was approved by the animal care committee of the University Of Calgary, Faculty of Medicine and conforms to the National Institutes of Health Guide for the Care and Use of Laboratory Animals. Eleven open chest mongrel dogs were anesthetized using 30 mg/kg of sodium pentobarbital administered intravenously, in addition to a maintenance dose of 75 mg/h for the length of the experiment. The dogs were intubated and mechanically ventilated (constant volume ventilator, model 607, Harvard Apparatus Company, Millis, MA, United States). The forelegs and left hind leg of the dogs were used for recording the ECG.

### Control Conditions

An ultrasonic flow probe (model T201, Transonic Systems Inc., Ithaca, NY, United States) was snug-fitted to the ascending aorta to measure blood flow rate, from which velocity (*U*) was derived using the *post mortem* diameter and assuming a circular cross-sectional area. Pressure at the aortic root (*P*) and LV (Plv) was measured using high-fidelity pressure catheters (Millar Instruments Inc., Houston, TX, United States). The aortic root catheter was placed approximately 1 cm distal to the aortic valve and was introduced into the aorta *via* the brachial or the carotid arteries. The LV pressure catheter was also inserted into either a brachial artery or into the LV directly through the myocardium of the LV apex. A mercury manometer was used to calibrate the pressure catheters before every experiment, and all data were digitally recorded at a sampling rate of 200 Hz. LV long and minor axes’ dimensions were measured throughout the cardiac cycle. Two pairs of ultrasound sonomicrometer crystals (5MH, Sonometrics, Ontario, Canada) were implanted in the mid-wall of the LV myocardium to measure the movement of the long (base–apex) and short (septum–free wall) axes throughout the cardiac cycle. The septum and free wall crystals were implanted at the mid-level, between the base and apex of the LV.

### Aortic Occlusion

Total aortic occlusion was achieved by a snare positioned in the proximal descending thoracic aorta at the level of the aortic valve approximately 13 cm from the measurement site. *P*, *U*, and axial shortening velocity were measured for 30 s during control and following aortic occlusion, 3 min after the snare was applied.

The following hemodynamic parameters were determined from the basic measurements: Pulse pressure (PP) = SBP − DBP, where SBP is the systolic blood pressure and DBP is the diastolic blood pressure. Mean arterial pressure (MAP) = DBP + (PP/3) and Stroke volume (SV) = (EDV − ESV), where EDV and ESV are end diastolic and systolic volume. Cardiac output (CO) = SV × heart rate (HR), where HR is the heart rate. Total peripheral resistance (TPR) = MAP/CO, and total arterial compliance (TAC) = SV/PP. Effective arterial elastance and compliance are, respectively, (EEA) = ESBP/SV, where ESBP is end systolic blood pressure, and (C) = SV/PP.

### Theoretical Analysis

Wave intensity analysis is based on the solution of the one-dimensional conservation equations of mass and momentum. Wave intensity (d*I*) is the rate of energy flux per unit area (W/m^2^) and can be written

(1) d⁢I=d⁢P⁢d⁢U.

The water hammer equation for forward (+) and backward (−) waves is

(2) $$d\,P_{\pm }=\pm \rho \,c\,d\,U_{\pm }$$

where ρ is the density of blood 1,050 kg/m^3^ and *c* is the wave speed, which we calculated using the PU loop, as previously described (Khir et al., 2001). Knowledge of *c* allows d*I* to be separated into its forward and backward components.

(3) $${\mathrm{dI}}_{\pm }=\pm \frac{1}{4\,\rho \,c}\,{\left(\mathrm{dP}\pm \rho \,\mathrm{cdU}\right)}^{2}$$

### Data Analysis

Custom-written programs in MATLAB (The MathWorks Inc., MA, United States) were used to analyze the data. *P* and *U* waveforms were smoothed using a 7-point Savitzky–Golay filter, and the foot of both waveforms was aligned to take account of time lags in data processing. Shifting by no more than three sampling intervals was required to adjust for the lag caused by the filter in the ultrasonic flow meter (Hollander, 1999). Up to three representative cardiac cycles were selected from a stable sequence of beats during the 30 s recording period. Each cardiac cycle was analyzed individually and the data were averaged for each dog.

The R wave of the QRS complex was taken as time *t* = 0 and the following hemodynamic events and their timings were identified: peak aortic pressure (*P*_max_); peak LV pressure (Plv_max_) and the derivative of Plv with respect to time ($\frac{d\,P\,l\,v}{d\,t}$); peak aortic velocity (*U*_max_); an inflection point on the descending limb of the aortic velocity waveform before aortic valve closure (*U*_*i*_) was determined from the first derivative of *U*_i_ with respect to *t*. LV volume was calculated by considering the LV as an ellipsoid using the following equation

(4) $$0.5\,\left(4/3\right)\times \pi \times r_{1}\,r_{2}^{2}.$$

Where *r*_1_ = LV long axis radius (base–apex) and *r*_2_ = septum to free wall radius whose dimensions were acquired using the sonomicrometer crystals. Time to peak volume decline (*V*_max_) was determined by plotting the first derivative of volume with respect to time.

**FIGURE 1:**
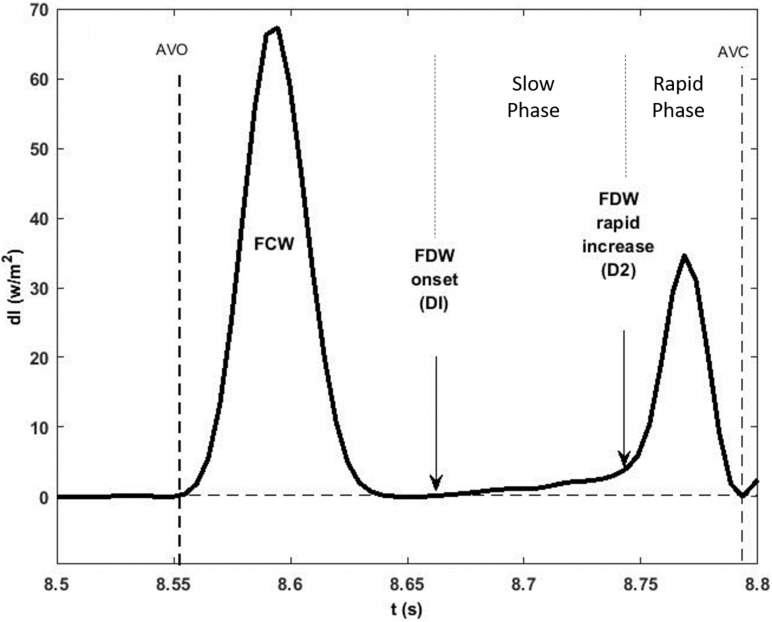
A typical forward wave intensity analysis curve calculated from pressure and velocity measured in the ascending aorta. The rise of the forward decompression wave, FDW, comprises two phases: a slow (D1) then a rapid (D2) phase whose onset is clearly shown to occur in mid and late systole, respectively. AVO and AVC indicate opening and closing of the aortic valve. Also shown is the forward compression wave, FCW, in early systole.

The onset of ejection was identified from the onset of the upstroke of the aortic flow waveform, which also indicated the onset of the FCW. The FDW wave observed in mid-to-late systole was always seen to have a “slow” (D1) then “rapid” (D2) phase as shown in Figure 1. The onset of each phase was determined from the first derivative of the forward wave intensity. The time of onset of the reflected waves was identified as the moment the separated backward wave intensity curve became negative. The energy carried by each wave (J/m^2^) was calculated by integrating the area under the peak of the wave with respect to time.

#### LV Wall Motion

**FIGURE 2:**
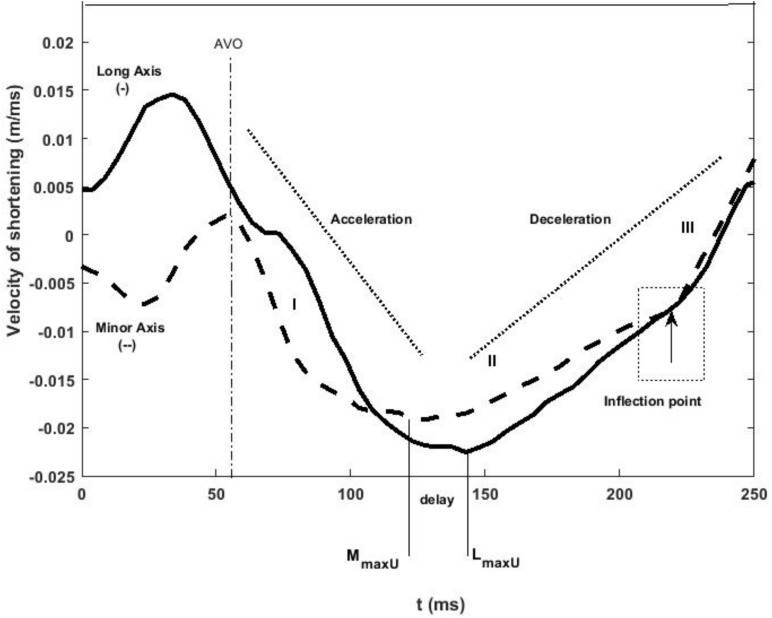
LV minor (dashed line) and long (solid line) axes shortening speed during systole. The minor axis reaches its maximum velocity of shortening before the long axis during control conditions. The three rates of axial shortening are represented by I, II, and III sequentially. In late systole, both axes exhibit an inflection point after which their velocity of shortening decreases at an increased rate indicated as rate III. AVO and AVC indicate aortic valve opening and closing, and stages I, II, and III indicate acceleration and deceleration of the axes at early, mid, and late systole, respectively.

Velocity of the LV long (*L*) and minor (*M*) axis shortening was determined by differentiating the axial displacement with respect to time. As reported previously (Page et al., 2010), the rate of shortening of each axis in systole could be subdivided into three phases throughout systole (I, II, III) although the onset of each phase in the long and minor axes did not occur simultaneously (Figure 2). Phase I represents acceleration of axis shortening during early systole; phase II is a period of slow deceleration of axial shortening beginning in mid-systole; phase III occurs near end-systole when there is a sudden increase in the rate of deceleration. The onsets of both phase II (*M*_maxU_, *L*_maxU_) and phase III (*M*_*III*_, *L*_*III*_) for each axis were determined from the second derivative of axial velocity of shortening. Minor and long axes rate of shortening (acceleration and deceleration) at each phase were also determined.

#### LV Wall Stress

Average fiber myocardial stress in the mid-wall (σ_*m*_) was estimated using the equations derived by Regen (1990).

(5) $$\sigma_{\text{m}=3/2\,\left({\text{V}}_{\text{m}}/{\text{V}}_{\text{mu}}\right)\,\text{P}/\left(\ln{\text{V}}_{\text{ou}}-{\text{lnV}}_{\text{cu}}\right)}.$$

Where *V*_*m*_ is mid-wall volume at any distension and *V*_*mu*_, *V*_*ou*_, and *V*_*cu*_ are mid-wall, chamber, and cavity volumes at reference distension. Based on the literature, end diastolic posterior wall thickness was assumed to be 7.1 mm in all dogs and was used to calculate LV wall volume. The average time to peak average fiber myocardial stress (σ_*max*_) was recorded.

### Reproducibility

Measurement of the time intervals from the peak of the R wave of the QRS complex to the peak shortening velocity of both the minor and long axes as well as to the onset of all three WIA waves was repeated on three separate occasions by a single observer. The mean difference ± SD to the time of *M*_maxU_ was 2 ± 2 ms (within-observer coefficient of variation (CV) = 2%). The time to *L*_maxU_ was 2 ± 4 ms (CV = 1%). The time to the onset of the FCW wave was 1 ± 2 ms (CV = 1.78%), the onset of the BCW was 1 ± 1 ms (CV = 1%), the onset of the FDW (D1) was 2 ± 3 ms (CV = 1%), and the onset of the FDW (D2) was 1 ± 4 ms (CV = 1%).

### Statistical Analysis

All data are presented as mean ± SD. Timings were compared by calculating the mean difference between timings in each dog and the standard deviation of the difference. Statistical agreement between timings was assessed using concordance correlation coefficients (CCC; asymptotic *p*-value) (Lin, 1989). All analyses were performed using Stata 12 (StataCorp) and *p* < 0.05 was considered statistically significant.

## Results

### Control Conditions

The baseline characteristics of the dogs during control are presented in Table 1.

**TABLE 1:** Baseline data during both control conditions and thoracic aortic occlusion.

**Variable**	**Control**	**Occlusion**	***p*-value**
SBP (mmHg)	116 ± 9	173 ± 9	<0.0001
DBP (mmHg)	73 ± 7	108 ± 7	0.003
MBP (mmHg)	88 ± 7	130 ± 8	0.001
PP (mmHg)	43 ± 5	65 ± 5	0.008
HR (bpm)	86 ± 5	90 ± 5	0.5
SV (ml)	13.0 ± 1.5	11.3 ± 1.7	0.5
CO (L/min)	1.2 ± 0.6	1.5 ± 0.8	0.3
TPR (kPa/L)	12.1 ± 6.7	13.6 ± 7.2	0.7
*C* (ml/kPa)	2.33 ± 0.6	1.38 ± 0.3	0.001
EEA (kPa/ml)	1.19 ± 0.4	1.92 ± 0.6	0.005
SEP (ms)	194 ± 11	174 ± 12	0.2
Peak *U* (m/s)	0.7 ± 0.09	0.5 ± 0.1	0.4

*SBP, systolic blood pressure; DBP, diastolic blood pressure; MBP, mean blood pressure; PP, pulse pressure; HR, heart rate; SV, stroke volume; CO, cardiac output; TPR, total peripheral resistance; C, arterial compliance; EEA, effective arterial elastance; SEP, systolic ejection period; peak U, peak velocity.*

**FIGURE 3:**
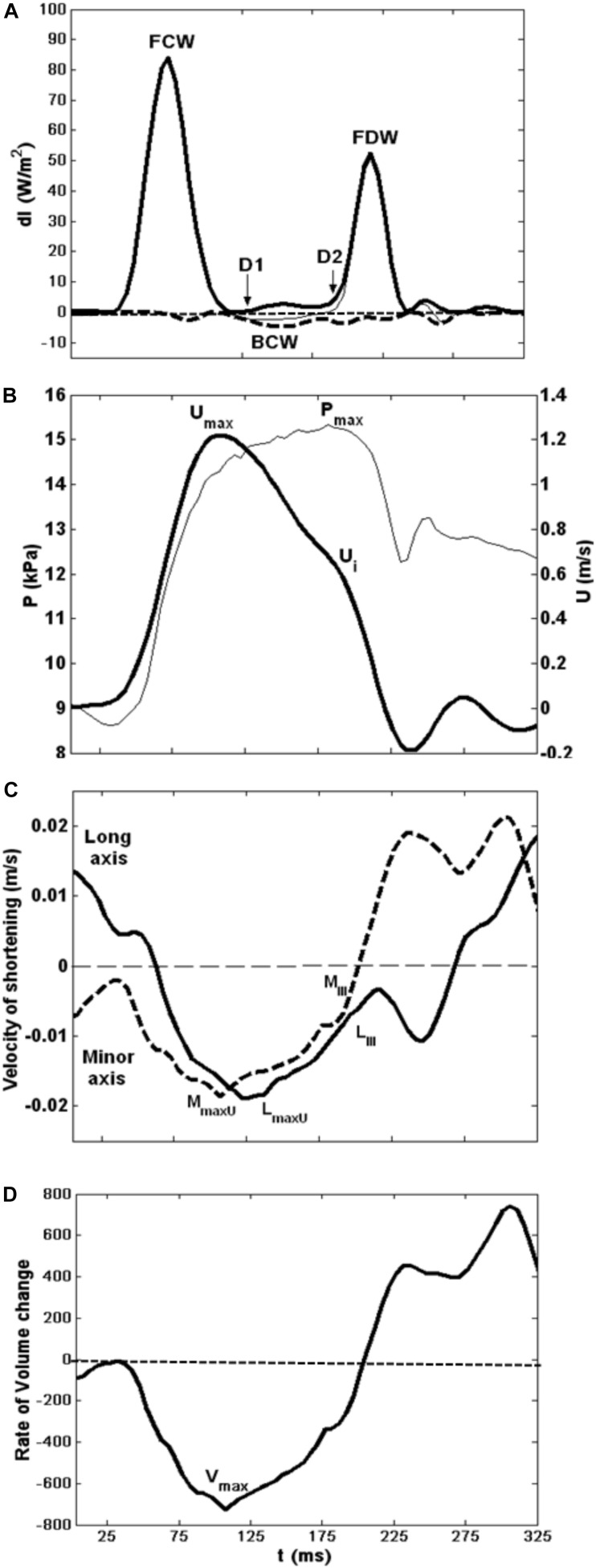
Composite showing all major hemodynamic events in sequence with velocity of LV shortening patterns. **(A)** Wave intensity analysis curves; forward waves (thick line). **(B)** Typical flow velocity waveform (thick line) and aortic pressure trace at the aortic root. **(C)** LV minor (dashed line) and long (solid line) axes shortening speed during systole. **(D)** LV rate of volume change.

**FIGURE 4:**
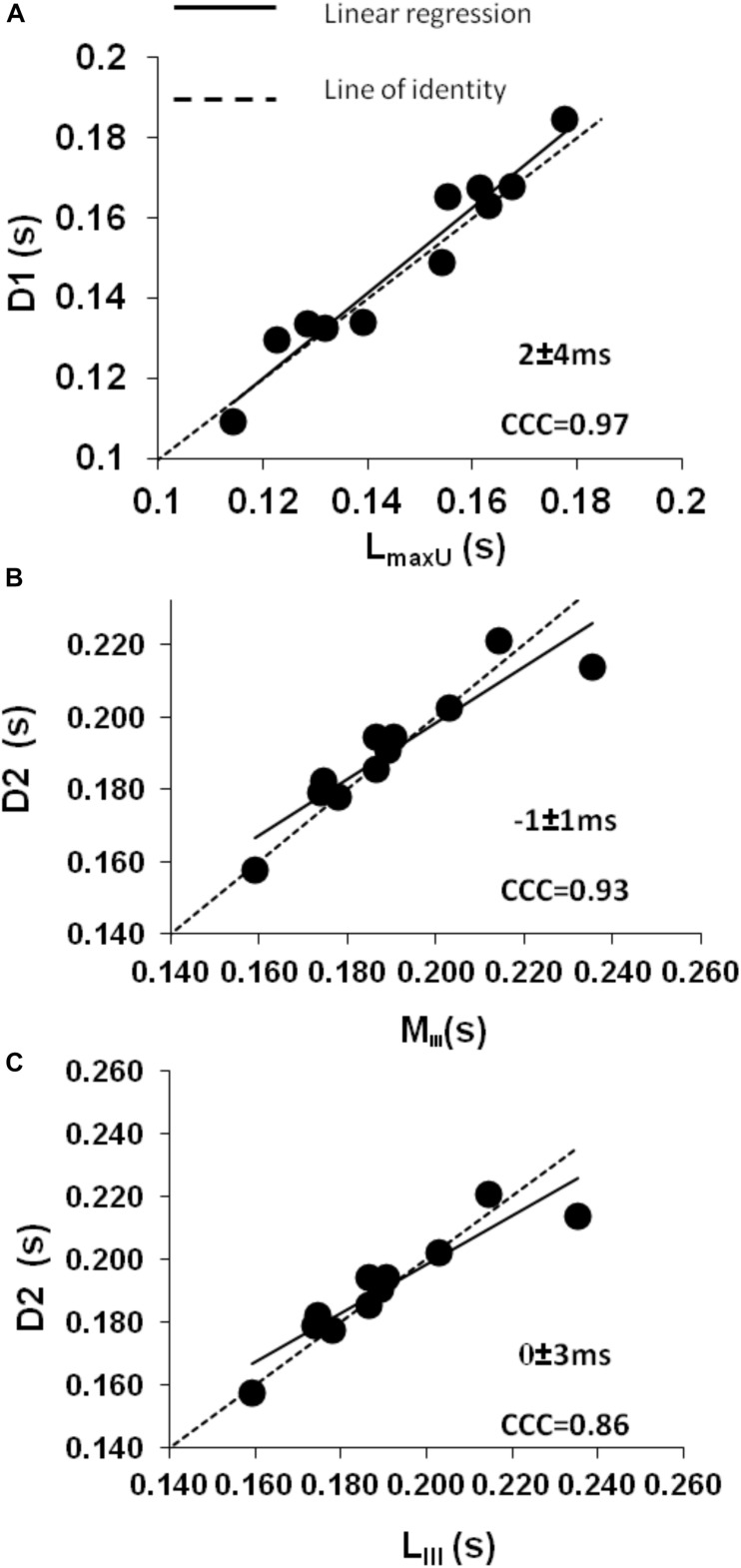
Control conditions: **(A)** intraclass concordance correlation plot (CCC) showing the strong agreement during mid-systole between the slow onset of the forward decompression wave (D1) and the time to maximum shortening velocity of the LV long axis (*L*_*maxU*_). **(B,C)** Intraclass concordance correlation plots (CCC) showing the strong agreement during late systole when a secondary decline in both **(B)** minor (*M*_I__I__I_) and **(C)** long axis (*L*_I__I__I_) shortening leads to the rapid phase of the forward decompression wave (D2) which in turn relates to a decrease in aortic pressure (*P*_*max*_) and a further decrease in the rate of declining velocity (*U*_*i*_).

The LV minor axis reached its maximum velocity of shortening, *M*_maxU_, at 110 ± 4 ms. The time of *M*_maxU_ agreed very closely with the arrival time of the reflected waves ($\bar{\text{x}}$ ± SD_diff_ = −3 ± 4 ms, CCC = 0.96, *p* < 0.001) (Table 2) and was shortly followed by *V*_max_ ($\bar{x}$ ± SD_diff_ = 4 ± 8 ms, CCC = 0.87). The long axis reached its maximum velocity of shortening, *L*_maxU_, approximately 40 ms after the minor axis (147 ± 20 ms) and after the time of arrival of the reflected wave. However, the time of *L*_maxU_ corresponded very closely with the time of the peak of the BCW (2 ± 7 ms, CCC = 0.91, *p* < 0.001) (Table 2). The onset of the decline in long axis velocity also corresponded with the onset time of the FDW (D1) ($\bar{x}$ ± SD_diff_ = 2 ± 4 ms, CCC = 0.97, *p* < 0.001; Figures 3, 4A). Around this time, σ_max_ also occurred (143 ± 32 ms after the R wave).

**TABLE 2:** All mean ± SD time intervals from the R of the QRS complex to the main hemodynamic and mechanical events during both control conditions and aortic occlusion.

	**Event**	**Control (ms)**	**Occlusion (ms)**
Minor axis maximum shortening velocity	*M*_*maxU*_	110 ± 4	93 ± 36
	BCW onset	106 ± 5*CCC*=0.96	97 ± 36*CCC*=0.96
	*V*_*max*_	117 ± 16*CCC*=0.87	103 ± 34*CCC*=0.97
Long axis maximum shortening velocity	*L*_*maxU*_	147 ± 20	158 ± 20
	FDW (D1)	149 ± 23*CCC*=0.97	153 ± 30*CCC*=0.69
	BCW peak	149 ± 22*CCC*=0.91	164 ± 15*CCC*=0.65
Rapid phase of the forward decompression wave (D2)	FDW (D2)	191 ± 18	201 ± 22
	*L*_I__I__I_	190 ± 20*CCC*=0.86	196 ± 28*CCC*=0.90
	*M*_I__I__I_	189 ± 20*CCC*=0.93	194 ± 25*CCC*=0.89
	*P*_*max*_	192 ± 27*CCC*=0.82	204 ± 21*CCC*=0.90
	*U*_*i*_	190 ± 17*CCC*=0.77	196 ± 27*CCC*=0.92
Wave energies (J/m^2^)	FCW	295 ± 198	232 ± 209
	BCW	−42 ± 13	−101 ± 24
	FDW	120 ± 57	78 ± 64

*CCC = concordance correlations. During both control and aortic occlusion conditions, the time of maximum shortening velocity of the LV minor axis (M_*maxU*_) coincides and has been compared with the time of the arrival of the BCW. The time of maximum shortening velocity of the LV Long axis (L_*maxU*_) coincides and has been compared with both the slow onset of the FDW (D1) and the peak of BCW. The rapid phase of the FDW (D2) coincides and has been compared to peak aortic pressure (P), an inflection point on the descending limb of aortic velocity (U_*i*_) and the change in rate of deceleration of the LV minor and long axis during late systole (L_Ø_ and M_Ø_). Plus average energies carried by the forward compression wave (S), reflected compression wave (R), and forward decompression wave (D) at control conditions and during total aortic occlusion. The forward waves are both attenuated during occlusion, while the reflected wave is significantly larger.*

**FIGURE 5:**
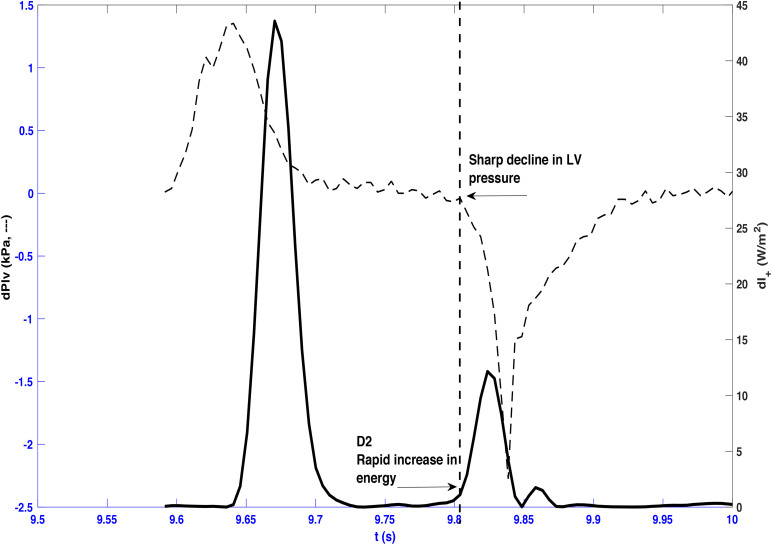
Forward wave intensity and differentiated left ventricle pressure (dPlv) plotted from a representative beat taken from dog 3. A sharp decline in left ventricle pressure coincides with the onset of the rapid increase of the forward decompression wave (D2) as indicated by the vertical dashed line.

A later marked decrease in the rate of LV axis shortening occurred almost simultaneously in the long and minor axes (190 ± 20 and 189 ± 20 ms, respectively). This was just prior to closure of the aortic valve (phase III) and corresponded very closely with the onset of D2 (long axis $\bar{\text{x}}$ ± SD_diff_ = 0 ± 3 ms, CCC = 0.86, *p* < 0.001; minor axis $\bar{\text{x}}$ ± SD_diff_ = −1 ± 1 ms, CCC = 0.93, *p* < 0.001) (Figures 4B,C). This time also coincided with the time of peak aortic pressure (*P*_max_) ($\bar{\text{x}}$ ± SD_diff_ = 1 ± 2 ms, CCC = 0.82, *p* < 0.001) and the occurrence of an inflection point (*U*_*i*_) on the descending limb of the aortic flow wave ($\bar{\text{x}}$ ± SD_diff_ = 0 ± 1 ms, CCC = 0.77, p < 0.001). As expected, D2 was also seen to coincide with a sharp decline in LV pressure ($\bar{\text{x}}$ ± SD_diff_ = 2 ± 2 ms, CCC = 0.9, *p* < 0.001) (Figure 5).

### Aortic Occlusion

To allow mechanistic links between wave reflection and LV shortening velocity to be elucidated, aortic clamping was used to alter the magnitude and timing of the reflected wave. During occlusion, there was a significant increase in systolic, diastolic, mean, and pulse pressure (Table 1), and this was accompanied by a significant reduction in arterial compliance but no significant change in heart rate.

**FIGURE 6:**
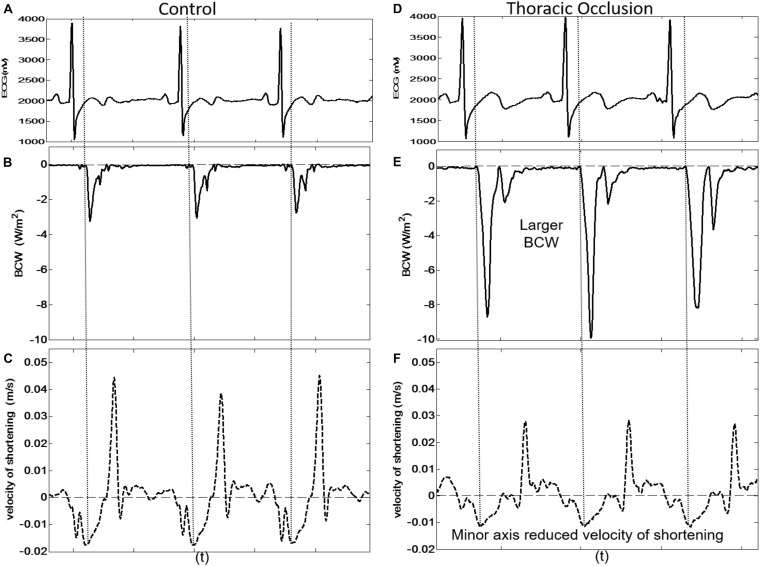
Three consecutive beats taken from dog 9 showing the simultaneous arrival of the BCW at the LV and reduced peak velocity of shortening of the LV minor axis during control conditions [**(A)** ECG, **(B)** BCW, **(C)** minor axis velocity of shortening] and during thoracic occlusion [**(D)** ECG, **(E)** BCW, **(F)** minor axis velocity of shortening].

Proximal aortic occlusion had a marked effect on wave reflection (Figure 6) increasing the energy of the reflected wave by more than twofold and resulting in an earlier arrival of the reflected wave (93 ± 36 ms, i.e., 12 ms earlier than control) although the time of the peak reflection was slightly delayed (164 ± 15 ms, i.e., 15 ms later than control). The FCW wave and FDW wave were reduced by 21 and 35%, respectively, by aortic clamping (Table 2). During aortic occlusion, *M*_maxU_ was reduced by 20% (from 1.5 ± 0.4 to 1.2 ± 0.2 m/s) and the time of *M*_maxU_ also occurred earlier (97 ± 36 ms); hence, the relationship between the arrival time of the reflected waves and the time of *M*_max__*U*_ was preserved ($\bar{\text{x}}$ ± SD_diff_ = 5 ± 9 ms, CCC = 0.96, *p* < 0.001) (Table 2 and Figure 6). During aortic occlusion, *V*_max_ still followed the time of *M*_maxU_ ($\bar{\text{x}}$ ± SD_diff_ 6 ± 6 ms, CCC = 0.97, *p* < 0.001), *L*_maxU_ reduced by 11% (from 0.9 ± 0.5 to 0.8 ± 0.2 m/s), and time of the peak reflected wave and *L*_maxU_ was in good agreement ($\bar{\text{x}}$ ± SD_diff_ = 4 ± 18 ms, CCC = 0.65, *p* < 0.001). This time also corresponded with the onset of FDW (D1) (Table 2). Aortic clamping had minimal effects on the timing of *L*_*III*_, *M*_*III*_, and D2 which occurred at approximately the same time (Table 2), and σ_max_ was significantly delayed compared to control conditions (200 ± 14 ms, *p* < 0.0001).

## Discussion

Waves carry important information about LV function and the interaction between LV and arterial circulation (Jones et al., 1993; Bleasdale et al., 2003). Our aims were to establish whether the arrival of reflected wave(s) affects LV long and minor axes shortening and to establish the relationship between LV shortening and the generation of the FDW in mid-late systole. The main findings are as follows: (1) arrival of reflected waves agrees very closely with the deceleration of the rate of shortening of the LV minor axis and rate of LV volume decline, even when the timing of the arrival of the reflected wave is altered by aortic clamping. This suggests that the arrival of the reflected wave initiates the deceleration of minor axis shortening; (2) the time of the peak of the reflected waves corresponds closely with the onset of deceleration of the rate of shortening of the long axis under control and aortic clamping conditions. This indicates that the reflected wave also plays a part in initiating the decline in deceleration in major axis shortening. (3) The FDW has a slow and then rapid phase, and these phases are explained by two distinct changes in the rate of shortening of the LV axes. The slow phase of the FDW (D1) is generated by deceleration of the LV long axis (i.e., its onset corresponds with the maximum velocity of shortening of the LV long axis), which in turn appears to be related to the peak of the reflected wave. The fast phase of the FDW (D2) is generated by concurrent deceleration of the rate of shortening in both long and minor axes.

### Wave Reflection and LV Wall Movement

The reflected wave is believed to play a detrimental role in the pathophysiology of cardiovascular disease in renal failure (London et al., 2001), hypertension (Manisty et al., 2010), and heart failure (Curtis et al., 2007) and to contribute to LV hypertrophy (Hashimoto et al., 2008). By employing aortic clamping as an intervention to cause a defined reflection site, we provide strong evidence for a causal link between arrival of the reflected wave, slowing of minor axis shortening, and peak volume rate of decline with a lesser effect on long axis function. This may have important clinical implications. In humans, long axis shortening is responsible for only 7% of ejection fraction in normal individuals but 18% in people with hypertension (de Simone et al., 1997). We speculate that the relative reduction in the contribution of the minor axis in systemic hypertension may be related to the adverse effects of earlier arrival and larger magnitude of reflected waves although it should be noted that the increase in magnitude of the reflected wave in hypertension is somewhat smaller than that following aortic occlusion (∼70% increase in backward pressure) (Merillon et al., 1983). Differences in long and minor axes’ sensitivity to reflected waves could result from differences in timing of the reflected wave in relation to the cross-bridge cycle in fibers of differing orientation. Previous studies have shown that there is a transition point in the cross-bridge cycle during systole; before this transition time, an increase in afterload prolongs contraction, whereas after this time, an increase in afterload abbreviates contraction (Brutsaert et al., 1985; Solomon et al., 1999). If the reflected wave arrives at the LV during the critical transitional time of minor axis contraction, this would promote cross-bridge detachment and early LV relaxation. Since in our study long axis contraction began after short axis contraction, it may be that this susceptible period occurs later in the cardiac cycle for the long axis. Further research on this issue is required.

### Left Ventricle Wall Motion and the Forward Decompression Wave

Sugawara et al. (1997) have previously proposed that a forward decompression wave is generated in late systole by the LV due to “both the loss of tension bearing ability caused by myocardial relaxation and the deceleration of aortic blood flow caused by the reduced rate of myocardial shortening.” This is analogous to the situation in isolated cardiac tissue, where contraction ceases before tension-bearing ability declines (Brutsaert and Sys, 1989). This explanation was supported by Niki et al. (1999) who showed that patients with mitral regurgitation had a much reduced FDW which increased following mitral valve surgery. Our data are consistent with this view, although they suggest a more complex pattern to the FDW in mid-late systole, with two distinct phases (D1 and D2). Jones et al. (2002) suggested that FDW may have been generated by two temporally distinct processes, such as the slowing of the myocardial shortening and complete cessation of myocardial shortening. We observed that D2 does not correspond to complete cessation of myocardial shortening but instead it coincides with a simultaneous decline in the velocity of shortening of both the minor and long axes later in systole. The mechanism responsible for the secondary change in velocity of deceleration of both LV axes is not known, but it is plausible that it corresponds to the release of torsion and radial thinning of the ventricle that is observed ∼40 ms before aortic valve closure (Rosen et al., 2004). The release of torsion would bring about a rapid decrease in LV pressure and a decrease in the rate of ejection resulting in a rapid increase in the intensity of the FDW (online Figure 4).

The strength of this study is the direct, invasive measurement of the reflected wave and left ventricular velocities. No assumptions about the timing and magnitude of waves were made, and measuring pressure and flow simultaneously to calculate wave intensity is superior to pressure waveform analysis alone. This work provides insight into how wave reflection influences LV function and wave generation. Wave reflection causes a deceleration in LV shortening and the function of the minor axis is particularly sensitive to the arrival of reflected waves. These observations lend support to previous suggestions that therapies directed toward reducing wave reflection may be of value in hypertension and cardiovascular disease.

A limitation of the study was that only longitudinal and radial motion were studied (i.e., base–apex and septum–free wall). Changes in LV geometry over the cardiac cycle are intrinsically complex, particularly during ejection, and possible effects of rotation could not be accounted for in our study. However, the crystals used for measuring the axial movement were embedded into the myocardium at the mid-level where rotation is minimal, and thus, this should not invalidate our principal conclusions. Nevertheless, further studies resolving the full three-dimensional strain patterns in the ventricle would be valuable. A further limitation is the lack of wall thickness measurements to investigate the effect of wave reflection on myocardial stress in more detail. A final limitation is the assumption that the aortic area is uniform throughout the cardiac cycle, and we cannot rule out the possibility of aortic distortion during the cross-clamping in or variability during the cardiac cycle (de Heer et al., 2011).

## Conclusion

In conclusion, This is the first study to simultaneously measure the arrival of reflected waves during systole with LV long and minor axes function. These measurements provided strong evidence that the arrival of the reflected wave initiates a deceleration in LV minor axis shortening. The data have also shown that the forward decompression wave generated by the LV has a slow and then rapid phase that are generated by two distinct changes in the rate of shortening of the LV axes. The results of this work are physiologically and clinically relevant as increased wave reflection causes a reduction in the contraction velocity of the LV. Reducing wave reflection may be of value in hypertension and cardiovascular disease patients.

## Data Availability Statement

The raw data supporting the conclusions of this article will be made available by the authors, without undue reservation.

## Ethics Statement

The animal study was reviewed and approved by animal care committee, Calgary University.

## Author Contributions

CP: data analysis, manuscript drafting, figures, and table production. AH: statistical analysis, data interpretation, and manuscript editing. MH: data interpretation and clinical implication and manuscript editing. AK: manuscript concept, data collection and interpretation and manuscript editing. All authors contributed to the article and approved the submitted version.

## Conflict of Interest

The authors declare that the research was conducted in the absence of any commercial or financial relationships that could be construed as a potential conflict of interest.
